# Sequential Therapy *vs* Quadruple Therapy for *Helicobacter pylori* Eradication in South West of Iran

**DOI:** 10.5005/jp-journals-10018-1103

**Published:** 2014-07-28

**Authors:** Abdol Rahim Masjedizadeh, Eskandar Hajiani, Seyed Jalal Hashemi, Pezhman Alavinejad, Hasan Dalvand

**Affiliations:** 1 Division of Gastroenterology and Hepatology, Department of Internal Medicine; Research Center for Infectious Diseases of Digestive System, Ahvaz Jundishapur University of Medical Sciences, Ahvaz, Iran; 2 Research Center for Infectious Diseases of Digestive System, Ahvaz Jundishapur University of Medical Sciences, Ahvaz, Iran

**Keywords:** *Helicobocter pylori*, Sequential therapy, Quadruple therapy, Eradication rate.

## Abstract

**Aim:**

To compare the efficacy of quadruple and sequential therapy in eradication of *Helicobocter pylori (H. pylori)* in a randomized study.

**Method:**

Three hundred *H. pylori* positive patients were enrolled into the study. These patients were randomly divided into two groups: group I (n = 150) received quadruple therapy (20 mg omeprazole bid, 240 mg bismuth subcitrate bid, 1,000 mg tetracycline bid and 500 mg metronidazole bid) for 14 days, group II (n = 150) received sequential therapy (20 mg omeprazole bid, 1,000 mg amoxicillin bid for 5 days, followed by 20 mg omeprazole bid, 500 mg metronidazole bid, 500 mg clarithromycin for the other 5 days). *H. pylori* status was assessed by histology and rapid urease test at baseline. Follow-up breath test by 14C urea breath test (UBT) was performed 4 weeks after completion of treatment. Eradication was defined as negative results on UBT.

**Results:**

Successful eradication was achieved in 245 patients. In each group, five patients did not tolerate the regimen and were excluded from analysis. About 29 (20%) patients who received sequential therapy and 21 (14.5%) of the quadruple group tolerated mild side effects (p = 0.21).

Per-protocol analysis demonstrated eradication rates of 86.9% for sequential therapy and 82.7% for quadruple therapy (p = 0.26). Results according to the intention to treat analysis were 84 and 79.5% in the sequential and quadruple group respectively. Eradication rate differences were not significant.

**Conclusion:**

The success rate of sequential therapy is comparable with quadruple therapy. Sequential therapy due to the short duration and lesser drug usage is a good alternative for eradication of *H. pylori* in the country.

**How to cite this article:** Masjedizadeh AR, Hajiani E, Hashemi SJ, Alavinejad P, Dalvand H. Sequential Therapy *vi* Quadruple Therapy for *Helicobocter pylori* Eradication in South West of Iran. Euroasian J Hepato-Gastroenterol 2014;4(2):63-66.

## INTRODUCTION

Therapeutic strategies for *Helicobacter pylori (H. pylori)* eradication vary in different regions of the world. In Western countries, triple therapy is considered as first line of therapy, while in Asian countries due to high-drug resistance, quadruple therapy is recommended.^1-3^

In Iran, quadruple therapy with the base furazolidone, tetracycline and clarithromycin as first line therapy is suggested.^4^ There are restrictions in therapy due to medical complications, the long duration of treatment and resistance to medications. Furazolidone causes side effects, such as nausea, vomiting and teratogenicity. A 57.5% resistance to metronidazole and 16.7% resistance to clarithromycin have been detected.^5^

Clarithromycin resistance reduced the eradication rate in the standard triple therapy. In communities with a higher than 15% resistance to clarithromycin, it is not recommended.^6^ Ten-day sequential therapy, including proton pump inhibitors (PPI) and amoxicillin for 5 days followed by clarithromycin and tinidazole in the next 5 days of treatment for *H. pylori* eradication in at least six trials in over 100 patients has been associated with eradication rates of more than 90%.^7^ In addition, in double-blinded and randomized studies, in comparison between sequential and PPI triple therapy, it has been associated with a greater eradication rate at an intention to treat (ITT) analysis of 91°% *vs* 78%.^8-14^ Moreover, sequential therapy was more effective in patients resistant to clarithromycin.^15^

There are few studies about the assessment of *H. pylori* eradication in sequential and quadruple therapies. The aim of this study was to compare quadruple therapy including omeprazole, tetracycline, metronidazole and bismuth for 14 days with a standard sequential therapy based on metronidazole instead of tinidazole for eradication of *H. pylori.*

## METHOD

Cases were selected from patients referred to outpatient clinic of Ahvaz Imam Hospital, Jundishapur Medical University, Iran, from February 2007 to December 2009. Inclusion criteria included patients older than 14 years of age with complain of dyspepsia, gastrointestinal bleeding and history of postprandial fullness or vomiting, who were performed upper endoscopy and two biopsies from the antrum and gastric body obtained. Those who had a positive rapid urease test or histological evidence for *H. pylori* in the gastric mucosa were enrolled. Patients with severe comorbidity, such as cirrhosis of liver, renal failure, severe heart disease, pregnancy, evidence of malignancy, a previous history of *H. pylori* eradication , a history of non-steroidal anti-inflammatory drug (NSAID) use, alcohol and H2 blocker or PPI consumption in the last 2 weeks or who had received antibiotics and bismuth subcitrate in the last month were excluded. Lactating mothers were also excluded from the study. Patients’ demographic characteristics, including age, gender, smoking and endoscopy results, were recorded. Patients with *H. pylori* infection were randomly divided into two treatment groups; namely, I and II. Group I received a 14 days course of therapy including 20 mg omeprazole bid, 1,000 mg tetracycline bid, 500 mg metronidazole bid and 240 mg bismuth subcitrate bid. Group II received sequential therapy including 20 mg omeprazole bid plus 1,000 mg amoxicillin for 5 days followed by 20 mg omeprazole, 500 mg clarithromycin bid and 500 mg metronidazole bid for the next 5 days.

## STATISTICAL ANALYSIS

Treatment success was evaluated per protocol and according to ITT analysis. The chi-square test and t-test were used for statistical analysis with significance at p <0.05. This study has registered in Iranian Registry of Clinical of Trials (IRCT) (IRCT201103183836N1).

## RESULTS

About 300 patients were included and divided into two groups of 150 cases. The mean ages in sequential and quadruple groups were 39.97 and 40.4 years respectively. In the sequential group, 77 patients were males (53%) and, in the quadruple group, 74 were males (51%) (Table 1). To ensure the comparison between groups regarding age and gender we used chi-square test for gender (p = 0.72) and t-test for age. No significant differences were observed statistically (p = 0.72).

We excluded five patients from the sequential group and five from the quadruple group due to complications and poor compliance. Thus, 145 patients from each group completed the study.

There was no statistical difference in the eradication rate between peptic ulcer disease (PUD) and non-ulcer disease (NUD) groups (p = 0.43 and 0.63 respectively), but in the gastritis patients, the rate of eradication between the two groups was significant (p = 0.045) (Table 2).

Based on ITT, the eradication rate was 84% in the sequential group and 79.3% in the quadruple group (p = 0.03). Pre-protocol (PP) rates in sequential and quadruple groups were 86.9% and 82.1%% respectively (p = 0.26) (Table 2).

**Table 1:** **Table 1:** Baseline characteristics of the study group (N = 300)

*Variable*		*Sequential*		*Quadruple*		*p-value*	
Mean age (SD) Y		39.97 (12.22)		40.48 (12.20)		0.72	
Male patients (%)		77 (53%)		74 (51%)		0.72	
Female patients (%)		68 (47%)		71 (49%)		0.72	
Smokers (%)		25 (17.2%)		17 (11%)		0.38	
*Endoscopy*							
NUD		38		35		0.68	
Gastritis		40		52		0.13	
DU		28		25		0.65	
Erosive gastritis		26		18		0.19	
Erosive duodenitis		5		9		0.27	
GU		4		4		1	
Duodenitis		5		3		0.72	

**Table 2:** **Table 2:** Eradication rates of *H. pylori* by quadruple therapy (PPI, bismuth, tetracycline and a nitroimidazole) as rescue therapy for triple therapy (PPI, clarithromycin and amoxicillin) failure

*Reference*		*Number of patients*		*Duration (d)*		*Eradication rate (%)*	
22		155		14		84	
23		87		7		84	
24		63		7		75	
25		70		14		63	
26		89		14		67	
27		100		14		82	
28		53		7		91	

The most frequent complication was change in taste, which reported by six cases in quadruple and nine cases in the sequential group. Other complications included anorexia, abdominal pain, nausea/vomiting, vertigo, diarrhea and skin rash, which were seen in 21 cases in the quadruple (14.5%) and 29 cases in the sequential group (20%).

In order to compare the complication rates, between the two treatment methods, we used Fisher’s test, and statistically no significant difference was obtained (p = 0.12).

All patients were visited again 1 and 2 weeks after starting of treatment for clinical assessment and to control how the drugs were used and to record the side effects of treatment. Patients were divided into three groups according to compliance; namely, good (uncomplicated and taking full course of treatment), average (despite complications, completed the treatment course) and poor (due to complication, the treatment was discontinued). Four weeks after completion of treatment, patients were evaluated by using carbon 14 UBT test for *H. pylori* eradication.

## DISCUSSION

Based on ITT analysis, at least 80% eradication rate and based on per protocol analysis at least 90% eradication rate is essential for *H. pylori* eradication.^16,17^ Quadruple therapy is an alternative treatment as first line treatment instead of triple therapy—PPI (clarithromycin and amoxi-cillin) in areas with high resistance to clarithromycin.^18^ So using this method, as the initial treatment strategy in country with the high resistance rate to clarithromycin seems logical. However, the use of sequential therapy with shorter duration and less medication could be a more suitable alternative. So far, 21 trials with sequential therapy have been published.^19^ Eradication rate of *H. pylori* in 2,058 of the 2,236 patients at ITT and PP analyses following sequential therapy were reported as 91.3% and 93.7% respectively. In these studies, the eradication rate had no association with the type of PPI, but the success rate changed according to the type of nitroimidazole used. In fact, the sequential therapy treatment using metronidazole instead of tinidazole has been associated with poor results. In a study which used metronidazole instead of tinidazole, *H. pylori* was eradicated in 301 of 358 patients and an 84.1% eradication rate was reported according to ITT.^20^ In other studies, the eradication rate at ITT analysis in 1,880 of about 1,930 patients treated with tinidazole was 97.4% (p < 0.001).^21^ These differences are due to the greater half-life of tinidazole compared with metronidazole. The study showed an 84% eradication rate based on ITT, which is lower than previous studies due to use of metronidazole instead of tinidazole. In Iran, tinidazole is more expensive and unavailable compared to metronidazole; therefore, metronidazole is prescribed in this treatment regimen.

Quadruple therapy (PPI, bismuth, tetracycline and metronidazole) as second line therapy after treatment failure of triple therapy (PPI, amoxicillin and clari-thromycin) as rescue regimen is recommended in several guidelines (Table 2). A 77% mean eradication rate has been mentioned for this regimen in a study by Gispert et al.^22^ Study with more samples and longer duration than previous studies with an ITT of 79.3% and a PP of 82.1% is close to the results of the mentioned studies. Comparison of two regimens according to age, gender and endo-scopic results were accomplished. The eradication rates showed no differences regarding age and gender. In the groups who underwent endoscopy and showed gastritis a significant difference in the eradication rate was detected between the groups who used quadruple therapy compared to the patients who used sequential therapy (p = 0.045). We assume that this was one of the limitations of the study causing bias due more samples of patients in the gastritis group.

## CONCLUSION

This study showed that sequential therapy as the first line therapy could be used in *H. pylori* eradication instead of the quadruple therapy in the country.
